# TKI耐药后针对T790M突变治疗

**DOI:** 10.3779/j.issn.1009-3419.2015.04.10

**Published:** 2015-04-20

**Authors:** 红 王, 瑞 郭, 力予 张

**Affiliations:** 100071 北京，中国人民解放军307医院肺部肿瘤科 Deputy Director of Lung Tumor Department, 307 Hospital, Beijing 100071, China

**Keywords:** 表皮生长因子受体, 酪氨酸激酶抑制剂, 肺肿瘤, TKI耐药, T790M, 阿法替尼, Dacomitinib, 厄洛替尼, 吉非替尼, Epidermal growth factor receptor, Tyrosine kinase inhibitors, Lung neoplasms, TKI resistance, T790M, Afatinib, Dacomitinib, Erlotinib, Gefitinib

## Abstract

表皮生长因子受体（epidermal growth factor receptor, EGFR）的口服活性小分子抑制剂的开发为非小细胞肺癌（non-small cell lung cancer, NSCLC）提供了新的治疗方案。EGFR基因发生激活突变的患者对EGFR酪氨酸激酶抑制剂（epidermal growth factor receptor tyrosine kinase inhibitors, EGFR-TKIs）治疗敏感，该疗法使大量患者临床获益。第一代可逆型ATP-竞争型EGFR-TKIs，吉非替尼和厄洛替尼作为一线、二线或维持疗法具有疗效。尽管这些药物具有初始疗效，但大多数患者在1年内会产生抗药性，50%-60%的患者都与T790M管家基因出现突变有关。新近的不可逆型EGFR-TKIs-阿法替尼和dacomitinib可共价结合并抑制多个ErbB家族的受体（EGFR、HER2和HER4）。人们主要评价这些药物作为一线治疗的意义，以及在对第一代EGFR-TKIs产生获得性抗药性情况下的意义。阿法替尼是获批的第一种ErbB家族阻断剂，用于治疗带有EGFR激活突变的NSCLC患者；dacomitinib正处在临床开发的后期阶段。特异性靶向T790M抗药性突变的EGFR抑制剂（AZD9291、CO-1686、HM61713）正处在早期开发阶段。正如本文中的讨论，EGFR-TKIs靶向的激酶范围不同，它们结合EGFR受体的可逆性和药物相互作用的潜能也不同。对于临床医生来说，这些差异对经多种药物治疗的NSCLC患者具有意义，从创新型抗癌药物联合治疗策略的角度看，这些差异也极具意义。

## Abstract (en)

**Background and objective:**

Epidermal growth factor receptor (EGFR) the development of orally activesmall molecule inhibitors for non-small cell lung cancer (NSCLC) provides anew treatment plan. EGFR gene mutation in patients with activation EGFR tyrosine kinase inhibitor (EGFR-TKIs) therapy for the treatment of sensitive, so that a large number of clinical benefit. The first generation of reversible ATP-competitive EGFR-TKIs, gefitinib and erlotinib as first-line, second-line or has the effect of maintenance therapy. Although the initial effect of these drugs have, but most patients will produce drug resistance. Within a year, 50%-60% patients had T790M housekeeping gene mutation associated with. Irreversible EGFR-TKIs recent background: afatinib and dac-omitinib covalent binding and inhibition of multiple ErbB family receptors (EGFR, HER2 and HER4). People evaluate these drugs as first-line treatment of significance, and acquired drug resistance situation significance on the first generation EGFR-TKIs. Afatinib is the first ErbB family approved blocking agent, used to treat with EGFR activatingmutations in patients with non small cell lung cancer; dacomitinib are in the later stages of clinicaldevelopment. EGFR inhibitors specifically targeting T790M resistance mutations (AZD9291, CO-1686, HM61713) are in the early stages of development. As discussed in this paper, the scope of the EGFR-TKIs kinase to target different, EGFR receptor binding was reversible and drug interaction potential is also different. For clinicians, these differences of the multi drug treatment of patients with non-small cell lung cancer with meaning, from the innovative anticancer drug combination therapy strategy point of view, these differences are also of great significance.

鉴定不同的激活突变对于定义新型靶向治疗方法非常关键^[1]^，这些激活突变可定义新的非小细胞肺癌（non-small cell lung cancer, NSCLC）的分子亚类。其中一个最著名的例子即为表皮生长因子受体（epidermal growth factor receptor, EGFR），在超过一半的NSCLC患者体内，这种细胞表面受体都被激活^[2]^。EGFR受体属于ErbB跨膜酪氨酸激酶受体家族，该家族包括EGFR（也称作ErbB1或HER1）、ErbB2（H-ER2）、ErbB3（HER3）和ErbB4（HER4）^[3]^。除了HER3以外，所有家族成员都具有酪氨酸激酶活性。EGFR/ErbB家族酪氨酸激酶受体对于细胞增殖、分化和凋亡都是必需的，因此可成为预防肿瘤生长和转移的有效靶点。

靶向EGFR的小分子酪氨酸激酶受体（tyrosine kinase receptor, TKIs）的研发使NSCLC的治疗发生了革命性变化。所谓的“第一代”EGFR-TKIs，厄洛替尼和吉非替尼可逆性地与三磷酸腺苷（adenosine triphosphate, ATP）竞争结合EGFR酪氨酸激酶的胞内催化域，因此，可以抑制EGFR的自磷酸化和下游信号^[4]^。在带有*EGFR*激活突变的肿瘤中，厄洛替尼和吉非替尼特别有效，10%-15%的白种人和40%的亚洲人NSCLC患者带有这种突变^[5]^。在90%的情况下，这些突变都为19号外显子缺失或21号外显子发生L858R替换^[5]^。

## 1 可逆型EGFR-TKIs

临床试验证明，与化疗相比，厄洛替尼和吉非替尼作为一线疗法，可明显改善晚期*EGFR*突变阳性的NSCLC患者的无进展生存期（progression free survival, PFS）和生命质量^[6-10]^。美国于2003年批准吉非替尼用作晚期NSCLC的三线疗法；但肺癌Iressa生存评价（ISEL）试验显示这两种药物对总生存期（overall survival, OS）不能产生获益，因此药物在新患者中的上市应用推迟到了2005年^[11]^。

2009年，欧洲批准吉非替尼用于带有*EGFR*突变的晚期NSCLC患者的全线治疗。厄洛替尼于2004年（美国）和2005年（欧洲）获批用于化疗抗性的晚期NSCLC的二线和三线治疗。2010年，厄洛替尼的用途又扩大到基于铂化疗后的维持治疗，然后又于2012年（欧洲）和2013年（美国）获批用于带有*EGFR*激活突变（19号外显子缺失或21号外显子发生L858R替换）的NSCLC的一线治疗^[12, 13]^。厄洛替尼和吉非替尼与EGFR的酪氨酸激酶结构域的结合是可逆的，因此这两种药物易受到影响ATP亲和力或激酶抑制剂本身的突变的影响。因此，尽管这两种药物的初始靶向治疗可产生良好的肿瘤应答，但*EGFR*突变阳性的患者经过9个月-12个月治疗后，最后总是会产生厄洛替尼或吉非替尼抗性^[6-10]^。其中一个重要的获得抗性的机制就是20号外显子中的管家基因*EGFR*出现了T790M突变，50%-60%的患者都会出现这种突变^[14, 15]^。该突变可提高ATP激酶的亲和力，从而降低抑制剂疗效^[15-17]^。此外，c-MET扩增、HER2扩增、小细胞转化和*PIK3CA*突变均与EGFR-TKI抗性的产生有关^[14, 15]^。因此，人们就需要开发在这种情况下具有疗效的新型靶向药物。

## 2 不可逆型ErbB家族阻断剂

不可逆结合EGFR受体、同时靶向多个ErbB家族成员的药物，包括在ErbB激活过程中起重要作用的HER2，也被描述为“第二代EGFR-TKIs”，可能会克服使用厄洛替尼和吉非替尼过程中观察到的药物抗性^[18]^。阿法替尼、dacomitinib和吉非替尼等不可逆EGFR-TKIs与ATP结合结构域的亲和力较高，可与ATP结合结构域形成不可逆的共价键，这些药物还可以抑制HER2，某些药物还可以抑制HER4。

阿法替尼是第一种获批用于带有*EGFR*突变的转移型NSCLC的一线治疗的可逆型ErbB家族阻断剂^[19, 20]^。LUX-Lung临床试验项目在对吉非替尼和厄洛替尼产生获得性抗性患者（LUX-Lung1、LUX-Lung4和LUX-Lung5）^[21-23]^进行二线或三线治疗，以及带有EGFR激活突变的患者（LUX-Lung2、LUX-Lung3和LUX-Lung6）进行一线治疗的背景中研究阿法替尼^[24-26]^。Ⅱb期/Ⅲ期LUX-Lung1显示用阿法替尼治疗两种化疗和厄洛替尼或吉非替尼均难治的患者可延长无进展生存期，但不会延长总生存期^[21]^。Ⅲ期LUX-Lung3和LUX-Lung6试验显示，与培美曲塞加顺铂（LUX-Lung3）或吉西他滨加顺铂（LUX-Lung6）相比，用阿法替尼治疗患有晚期肺癌并带有*EGFR*突变的未致敏患者，可延长无进展生存期，并改善肿瘤相关的症状和总体健康状况^[25-29]^。美国、台湾和欧洲于2013年批准阿法替尼用于带有*EGFR*激活突变的转移型NSCLC的一线治疗，LUX-Lung3^[25]^为此提供了基础^[19, 20]^。这两项试验的汇集分析的初步结果显示，与标准化疗相比，带有常见*EGFR*突变（19/L858R缺失）的患者用阿法替尼治疗可改善总生存期（27.3个月-24.3个月；风险比=0.81，*P*=0.037）^[27]^。在LUX-Lung3试验中的19号外显子（33.3个月*vs* 21.1个月）发生缺失突变的肿瘤患者中，这一效果更加明显[风险比=0.54]；在LUX-Lung6试验中，结果分别为31.4个月和18.4个月[风险比=0.64]。此外，Ⅱ期试验证明，在多种情况下，包括带有EGFR突变型肿瘤或已知的T790M突变^[30]^的患者，以及化疗和TKIs难治型患者^[31]^用一种或两种化疗方案治疗失败，以及用厄洛替尼作为一线治疗失败，另一种不可逆型EGFR-TKI，即dacomitinib（PF-00299804）具有益处。Ⅲ期试验比较了既往进行化疗（二线/三线）的晚期NSCLC患者使用dacomitinib和厄洛替尼（ARCHER 1009）的疗效，或者比较了TKI和化疗失败后使用dacomitinib和安慰剂的疗效^[32, 33]^。ARCHER1009试验未达到dacomitinib比厄洛替尼能更好改善无进展生存期的目标；BR.26试验也未能证明与安慰剂相比（正如今年ASCO摘要8018Ellis PM报告的NCIC CTG BR.26研究结论：经治的晚期NSCLC中，未显示更好的活性)，dacomitinib可显著延长总生存期。一项进一步的Ⅲ期试验比较了dacomitinib与厄洛替尼在带有*EGFR*突变型肿瘤的未治疗患者中的疗效，试验正在进行过程中，预计于2015年得到结果。此外，在患有NSCLC，且对第一代EGFR-TKIs产生过反应的患者，以及未进行TKI治疗的患者中，临床试验正在验证来那替尼的疗效^[34]^，但由于反应率较低，以及出现了剂量限制性腹泻，单一疗法治疗评价被中断。在实体瘤患者中，观察到来那替尼和西罗莫司脂化（一种mTOR抑制剂）联合治疗可产生益处^[35]^。因此，现在正在评价带有HER2突变的NSCLC患者联合使用来那替尼和每周使用西罗莫司脂化的疗效^[36]^，考虑到缺少益处，以及来那替尼单一疗法的未来临床发展，本文未进一步讨论该药物。

## 3 突变选择型EGFR-TIKs

所谓的更新的“第三代”EGFR-TKIs，靶向EGFR激活突变和T790M，但不靶向野生型EGFR，现在也正在研发其作为一线治疗。三种此类化合物，即AZ-D9291、CO-1686和HM61713均为靶向EGFR激活和抗性（T790M）突变的口服、不可逆、选择性抑制剂^[37-40]^。正在进行的Ⅱ期剂量增加试验显示，在EGFR突变型NSCLC（主要带有T790M）和对既往EGFR-TKI治疗产生抗性的患者中，肿瘤出现了明显缩小^[41-43]^。不靶向正常皮肤和肠道细胞中的野生型EGFR被认为与治疗指数出现改善有关。2014年计划实施一个广泛的Ⅱ期/Ⅲ期研发项目，该项目使用C-O-1686（TIGER Ⅰ-Ⅴ试验）作为因T790M突变而对EGFR靶向疗法产生获得性抗性的NSCLC患者的二线疗法，以及EGFR突变型肿瘤患者的一线疗法。AZD9291和HM61713的进一步研发还未得到官方宣布。

## 4 受体活性的特性

所有EGFR-TKIs均对EGFR受体有高亲和力^[44-47]^；阿法替尼和dacomitinib对HER2和H-ER4受体也具有高亲和力。阿法替尼还可抑制HER3的反式磷酸化，从而阻断所有ErbB家族成员的信号^[4-5]^。与吉非替尼和厄洛替尼相比，阿法替尼在带有*EGFR*激活突变的细胞中的体外活性更好，在多种癌症异体移植模型中的抗肿瘤活性更好，其中包括对现在上市的EGFR抑制剂产生抗性或表达T790M的*EGFR*突变型细胞系，在动物模型中的抗肿瘤活性也更好^[45, 46]^。临床前研究^[47, 48]^显示，dacomitinib可抑制多种*EGFR*突变体，包括常见的激活突变和T790M突变体，还可有效减少吉非替尼抗性的非小细胞异种抑制物的生长。

突变体选择性EGFR-TKIs均对突变型*EGFR*受体具有高选择性，包括激活突变和T790M突变^[37]^。但对野生型EGFR选择性较弱^[37-39]^。在体外研究中，突变体选择性EGFR-TKIs均可有效抑制表达L858R EGFR或L858R/T790M EGFR的NSCLC细胞系的增殖，但对野生型EGFR细胞系的活性较弱。与之类似，体内研究表明，在不同的EGFR突变型细胞系异种抑制模型中，突变体选择性EGFR-TKIs可以剂量依赖的方式抑制肿瘤生长^[37, 38, 40]^。

总之，与厄洛替尼或吉非替尼相比，阿法替尼和dacomitinib具有更广泛的抑制活性，包括HER2和H-ER4受体，以及厄洛替尼或吉非替尼抗性的NSCLC异种移植物。尽管阿法替尼和dacomitinib在具有T790M依赖的获得性抗性情况下可能具有体外活性，但其临床益处有限^[21, 28]^。这是与野生型EGFR抑制有关的剂量限制性毒性导致的结果，因此阿法替尼和dacomitinib的剂量不足以抑制T790M药物抗性突变^[49, 50]^。在这种情况下，突变体选择性EGFR-TKIs或双靶向EGFR（例如阿法替尼和西妥昔单抗联合用药）的治疗前景最好。

## 5 结论

总之，EGFR-TKIs提供了治疗NSCLC的针对性方法。对吉非替尼、厄洛替尼、阿法替尼和dacomitinib的回顾分析突出了他们在各方面的差异，这种差异会影响他们在药物与药物的相互作用中的潜能，在使用多种药物治疗的癌症患者的确定中是高度相关的。在日常临床实践中，阿法替尼可能提供一些理论上的优势，尤其是缺乏细胞色素相关互相作用的潜能以及与酸减少药物（H2受体拮抗剂，质子泵抑制剂和抗酸药）互相作用的潜能。汇集的阿法替尼LUX-Lung3和LUX-Lung6生存数据的明确统计分析和相关出版物在等待中。未来突变体选择性EGFR-TKIs如CO-1686、AZD9291和HM61713，可能为患者提供潜在临床前景，从而防止观察到吉非替尼和厄洛替尼耐药性的出现，从而改善患者的无进展生存期。
